# Large Fragment Pre-S Deletion and High Viral Load Independently Predict Hepatitis B Relapse after Liver Transplantation

**DOI:** 10.1371/journal.pone.0032189

**Published:** 2012-02-21

**Authors:** Ting-Jung Wu, Tse-Ching Chen, Frank Wang, Kun-Ming Chan, Ruey-Shyang Soong, Hong-Shiue Chou, Wei-Chen Lee, Chau-Ting Yeh

**Affiliations:** 1 Division of Liver and Transplantation Surgery, Department of General Surgery, Chang Gung Memorial Hospital, Taoyuan, Taiwan; 2 Graduate Institute of Clinical Medical Sciences, Chang Gung University, Taoyuan, Taiwan; 3 Department of Pathology, Chang Gung Memorial Hospital, Taoyuan, Taiwan; 4 Liver Research Center, Department of Hepato-Gastroenterology, Chang Gung Medical Center, Taoyuan, Taiwan; University Hospital of Essen, Germany

## Abstract

Hepatitis B virus (HBV) associated end-stage liver diseases are the leading causes of liver transplantation (LT) in Taiwan. Relapse of hepatitis B occurs after LT, raising the risk of graft failure and reducing patient survival. Although several oral antiviral agents have been approved for anti-HBV treatment, lamivudine (LAM) remained to be the most widely used preventive regimen in Taiwan. While several clinical predictors have been identified for hepatitis B relapse, the predictive roles of the histopathological characteristics in liver explants as well as the genotypic features of the viruses in pre-LT serum samples have not been assessed. Between September 2002 and August 2009, 150 consecutive hepatitis B surface antigen (HBsAg) positive patients undergoing LT were included for outcome analysis following assessment of the clinicopathological and virological factors prior to LT. Kaplan-Meier analyses discovered that pre-operative LAM treatment ≤3 months; membranous distribution and higher expression of tissue HBsAg in liver explants; preoperative viral load ≧10^6^ copies/ml; and presence of large fragment (>100 base pairs) pre-S deletion (LFpreSDel) correlated significantly with hepatitis B relapse. Multivariate Cox regression analysis showed that the presence of LFpreSDel (*P* = 0.001) and viral load ≧10^6^ copies/mL (*P* = 0.023) were independent predictors for hepatitis B relapse. In conclusion, besides high viral load, LFpreSDel mutation is an important independent predictor for hepatitis B relapse after LT. More aggressive preventive strategies should be applied for patients carrying these risk factors.

## Introduction

Hepatitis B virus (HBV) infection remains a major public health problem worldwide with approximately 2 billion people having been infected and 350 million of these patients becoming chronic carriers [1]. HBV infection is endemic in Taiwan and the carrier rate of HBsAg in general population has been as high as 15–20% before initiation of the vaccination program. Infection of HBV is associated with a wide spectrum of clinical manifestations, ranging from acute or fulminant hepatitis to various forms of chronic diseases, including asymptomatic carriers, chronic hepatitis, liver cirrhosis, and hepatocellular carcinoma (HCC) [1], [2]. Since the first successful human liver transplantation (LT) in 1963 by Starzl et al [3], LT has become the treatment of choice in eligible patients with hepatic failure or early HCC in many medical centers. However, the results of early experience with LT for HBV related diseases were unsatisfactory in the 1980s, as majority of patients developed recurrent HBV infection in the early post-LT period, leading to loss of the allograft with reduced patient survival [4], [5]. Patients with active viral replication prior to transplantation are particularly at risk of hepatitis B relapse. On the other hand, in patients harboring a lower viral load prior to LT, a favorable long-term outcome is generally expected. This observation led to an effective strategy to improve LT outcome through reduction of pre-transplantation HBV viral load [6].

The clinical outcomes of patients as well as graft survivals in HBV LT recipients have been dramatically improved with the introduction of long-term hepatitis B immune globulin (HBIG) therapy and the availability of highly effective antiviral agents such as lamivudine (LAM) and adefovir dipivoxil [7]. Furthermore, combination prophylaxis with the use of HBIG and antiviral agents has reduced the risk of hepatitis B relapse to 10% or less in the first 2 years following transplantation [8], [9], [10], [11], [12]. As a result, the outcomes of patients with acute and chronic HBV related liver diseases undergoing LT are now similar to or better than those with non-HBV related liver transplantation [13], [14]. While the prophylactic therapies in the management of HBV in transplant recipients represent a significant step in LT, there remain controversies and challenges that have not been adequately resolved. Long-term prophylaxis with HBIG is expensive and has been associated with the development of surface antigen mutations. Similarly, emergence of drug resistance caused by tyrosine-methionine-aspartate-aspartate (YMDD) motif mutants occurs with prolonged LAM therapy. The choice of antiviral drugs or drug combinations and duration of prophylaxis are still being debated in the literature. It has been suggested that short-term rather than life-long HBIG could be used with or without combination of oral nucleos(t)ide analogs in low risk patients [15], [16], [17]. This strategy emphasized the need to identify risk factors capable of predicting hepatitis B relapse after LT for optimal prophylaxis. While several studies have identified clinical predictors such as high viral loads [18], [19], cumulative corticosteroid dose for immunosuppression [20], recurrence of HCC post LT [21], [22], HBIG monoprophylaxis (12) [12] and prolonged LAM therapy [19], [23], none has assessed the predictive roles of the histopathological characteristics in liver explants as well as the genotypic features of the viruses in pre-LT serum samples. The aim of this study, therefore, was to evaluate the predictive value of all the aforementioned clinicopathological and genotypic virological factors in hepatitis B relapse after LT in patients receiving combination treatment of short-term HBIG and life-long LAM.

## Methods

### Ethics statement

This study was conducted under the approval of the Institutional Review Board, Chang Gung Memorial Hospital, Taiwan. Written informed consent was obtained from all patients included.

### Patients

Between September 2002 and August 2009, 150 consecutive HBsAg positive patients undergoing LT in Chang Gung Memorial Hospital Linko medical center were included under informed consent. The indications for LT in this group of patients included HCC, decompensated liver diseases and fulminant hepatic failure. All patients were alive more than 3 months after LT and were followed up monthly in the outpatient clinic. This study was approved by the institutional review board at Chang Gung Memorial Hospital.

### HBV prophylaxis protocol

Prior to transplantation, LAM (Zeffix, GlaxoSmithKline, Middlesex, UK) (100 mg/day orally) was commenced in 79 patients. Among them, 39 patients received the prophylactic therapy for more than one month. Eight patients were confirmed to develop YMDD mutation at transplantation, and subsequently received adefovir (Hepsera, GlaxoSmithKline, Middlesex, UK) monotherapy. During LT operation, intravenous HBIG (Hepatect, Biotest Pharma, Dreieich, Germany) (10,000 IU) was administered in the anhepatic phase, followed by 2,000 IU daily after LT for one week. All patients received daily oral LAM (n = 142) or adefovir (n = 8) antiviral therapy after LT.

### Virological assays for HBV

Prior to transplantation, viral markers including HBsAg, antibody against hepatitis B surface antigen (anti-HBs), hepatitis B e antigen (HBeAg), antibody against hepatitis B e antigen (anti-HBe), antibody against hepatitis B core antigen (anti-HBc) and antibody against hepatitis C virus (anti-HCV) were routinely measured using commercially available assays for the donors and recipients. The sources of these standard tests were described previously [24]. Serum viral DNA was extracted using commercial kits (QIAamp DNA Blood and Tissue Mini Kit; QIAGEN Inc., Valencia, CA). The HBV-DNA concentration was quantified using Roche TaqMan HBV Monitor (Roche Diagnostics, Basel, Switzerland) with a detection limit of 69 copies/mL. The preoperative serum samples were used for virological analyses including viral genotypes, large and short fragments pre-S deletions, precore G1896A mutation and basal core promoter A1762T/G1764A mutations. The methods of these genotypic assays were described previously [24]. Following LT, liver function profiles were monitored daily for the first week, weekly for the first month, and monthly thereafter. HBV serological markers were monitored weekly for the first month and monthly thereafter. Hepatitis B recurrence was defined as reappearance of HBsAg or HBV-DNA in the serum. Experiments were conducted to identify LAM or adefovir resistance-associated viral mutations in patients with recurrent hepatitis B.

### Immunosuppression

The immunosuppressive agents including calcineurin inhibitors and corticosteroids were administered for all eligible patients after LT. No intraoperative high dose methylprednisolone (Solu-medrol, Pfizer, New York City, NY) was given in our medical center. The corticosteroids were gradually tapered over 3 months after LT. Oral tacrolimus (PROGRAFT, Astellas, Tokyo, Japan) was commenced 48 hours after transplantation and the dose was adjusted to maintain serum trough levels over 5–10 ng/ml. Alternatively, mycophenolate mofetil (CellCept, Roche, Basel, Switzerland) was administered in selected patients with renal insufficiency. A liver biopsy was performed if acute cellular rejection was suspected clinically or biochemically. In case of acute graft rejection, an intravenous bolus dose (500 mg) of methylprednisolone was administered for 1–3 days, followed by rapid weaning of intravenous corticosteroid over 5–7 days. Oral prednisolone was slowly tapered for 3–6 months after the pulsed steroid therapy was administered.

### Histopathology analysis of liver explants

Four specimens were randomly sampled from sections of explanted liver for histopathological analyses using hematoxylin and eosin staining. The Ishak histology score was used to assess the severity of hepatitis [25]. Immunohistochemistry for HBsAg and HBcAg were performed on formalin fixed paraffin embedded tissues using mouse monoclonal antibodies (Clone 1044/341 and LF161, respectively, Abnova Corporation, Taipei, Taiwan). The tissue blocks from each explanted liver were sectioned at 3 µm and placed onto positively charged slides. Slides were then stained using the Bond-Max autostainer (Leica Microsystems Inc., Buffalo Grove, IL) according to the manufacturer's protocol. Antibody detection was performed using the biotin free Bond Polymer refined Detection System (Leica Microsystems). Slides were counterstained with hematoxylin. The percentage of chromogen containing cells were estimated in the ranges of 0%, <5%, 5%–25%, 26%–50%, >51% and semiquantitively scored as 0 to 4. The characteristics of HBsAg stain were further classified into cytoplasmic or membranous expression. The characteristics of HBcAg stain were classified into nuclear or cytoplasmic expression. Histopathological assessment was performed by pathologists who specialized in liver pathology and was blinded to the clinical and virological data.

### Statistical analysis

Fisher's exact and Pearson's χ^2^ tests were used, where appropriate, to compared dichotomous variables. Student's t test was used to compare parametric data. Kaplan-Meier method was used to compare the overall survivals or hepatitis B relapse free survivals between subgroups. In the survival analysis, the cutoffs of parametric data were obtained following a published procedure [24]. Statistical differences in survival analysis were calculated using the log rank test. Factors with *P*<0.05 in Kaplan-Meier analysis were included for multivariate analysis using stepwise Cox proportional hazard model. All statistical analyses were performed using SPSS version 13.0 (SPSS, Chicago, Illinois, USA).

## Results

### Clinical characteristics of the patients and transplantation outcomes

**Figure 1:**
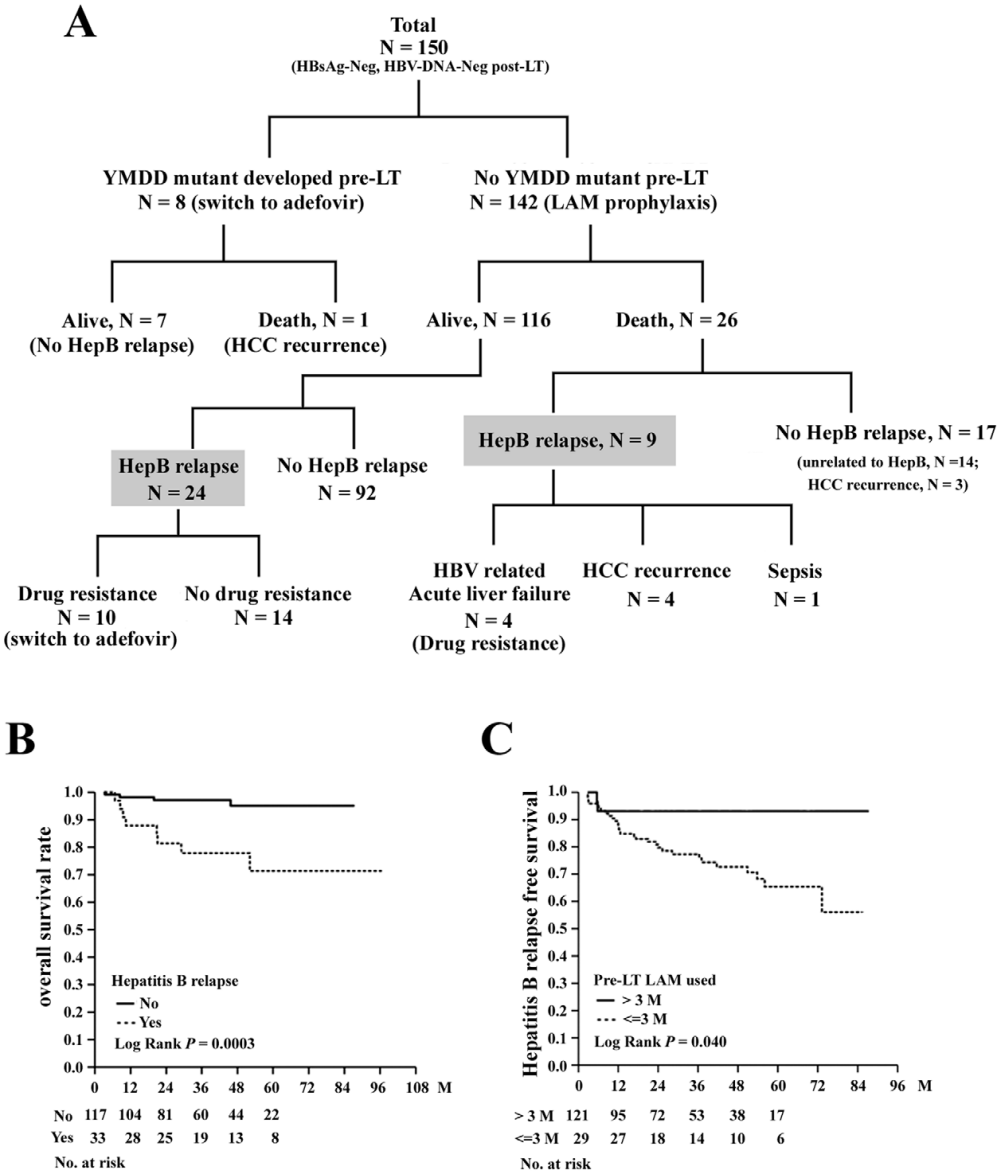
Clinical outcomes of the patients included and clinical factors related to OS or hepatitis B relapse free survival. (A) Clinical outcomes of 150 patients receiving LT. Hepatitis B (HepB) relapse did not occur in 8 patients who developed YMDD mutants and were switched to adefovir therapy before LT (left branch), whereas HepB relapse occurred in the remaining 142 patients who did not develop YMDD mutants. The clinical consequences of these patients were illustrated. The 33 patients with hepatitis B relapse were marked by a shaded background. (B) Comparison of OS between patients with (dashed line) and without (solid line) hepatitis B relapses. (C) Comparison of hepatitis B relapse free survival between patients receiving longer (>3 months; solid line) and shorter (≤3 months; dashed line) durations of pre-LT LAM treatment.

The baseline clinical parameters and operation associated variables were listed in Table S1. The immunosuppressant used and occurrence of infections were listed in Table S2. Of the 150 patients included, 123 (84.0%) received LT because of advanced liver cirrhosis with or without HCC, 20 (13.3%) because of acute on chronic liver failure and 7 (4.7%) because of fulminant hepatitis. HCC was found in the explanted livers in 78 (52.0%) patients. The median follow-up period of the 150 patients was 37.5 months (range, 3.2–96.6). The patient's outcomes were shown in Figure 1A. All patients were tested negative for HBsAg and HBV-DNA immediately after LT. In this study, reappearance of HBsAg or serum HBV-DNA was considered hepatitis B relapse. As such, 33 patients (22.2%) experienced reappearance of HBsAg during the follow-ups. Of them, 18 (12.0%) patients also experienced reappearance of serum HBV-DNA. Most of these patients (15 of 18 patients) with re-appearance of viremia after LT were found to develop LAM-resistant (YMDD) mutation and add-on Adefovir were given as anti-viral therapy. None of our patients was positive for serum HBV-DNA but negative for HBsAg. The median time to hepatitis B relapse was 12.2 months (range, 2.8–73.2). The cumulative hepatitis B relapse rates were 10.5% in the first year, 19.6% in the third year, and 29.7% in the fifth year, respectively. Twenty-seven (18%) patients died. Of them, 8 died of recurrent HCC, 4 died of acute liver failure due to hepatitis B relapse and the other 13 patients died of non-HBV related diseases including sepsis and biliary complications. The median time to death after LT was 8.4 months (range, 3.2–72.6) in these patients. Kaplan-Meier analysis showed that hepatitis B relapse was significantly associated with shorter overall survival (*P* = 0.0003; Figure 1B).

### Clinical factors associated with hepatitis B relapse after LT

The baseline clinical parameters and operation associated variables were compared between patients with (n = 33) and without (n = 117) hepatitis B relapse (Table S3). Kaplan-Meier analysis indicated that only preoperative LAM treatment ≤3 months was significantly associated with shorter hepatitis B relapse free survival (*P* = 0.040) (Figure 1C). In 29 patients receiving LAM treatment >3 months prior to LT, 3 (10.3%) had a viral load >10^6^ copies/mL at the time of LT. In comparison, 36 of 121 (29.8%) patients receiving LAM treatment ≤3 months had a viral load >10^6^ copies/mL (*P* = 0.035).

The information of steroid and immunosuppressant usages as well as treatment of acute rejection with steroid bolus was given in Table S2. There were ten patients suffered from acute rejections within half year before the date of HBV relapse or last follow-up. We did not use anti-IL2 receptor monoclonal antibody for treatment of acute rejection. Episodes of infections by bacteria, fungus, or cytomegalovirus were also listed in Table S2. None of these factors were associated with HBV relapse.

There were 52 LT patients receiving a graft from Anti-HBc positive donors (See Table S1). Seven of these 52 patients had HBV relapse. The donors of these 7 patients were negative for HBsAg and positive for anti-HBs antibody. It was difficult to prove that the reactivation was donor derived. However, it was interesting to note that one patient with HBV relapse had altered HBV genotypes before operation and after relapse (C to B). This patient might have a donor derived hepatitis B reactivation.

### Virological and histopathological factors associated with hepatitis B relapse after LT

**Figure 2:**
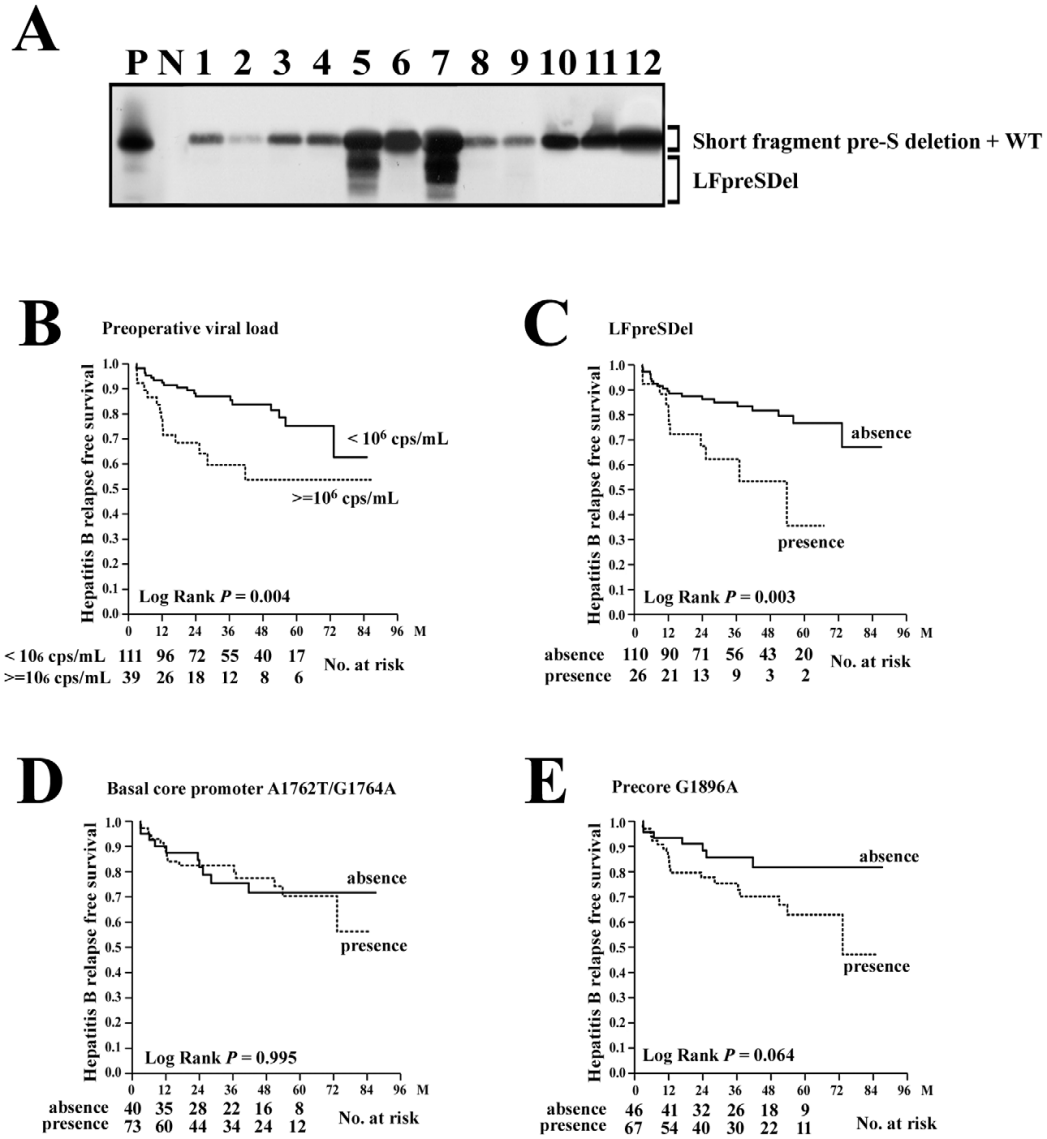
Association between hepatitis B relapse free survival and the genotypic features of HBV. (A) Detection of the LFpreSDel mutants. After PCR amplification of the HBV pre-S sequence, the product was submitted for Southern blot analysis using a specific probe. N, 15 pg of pCR2.1-TOPO vector DNA as a negative hybridization control; P, 15 pg of positive PCR product as a positive control; WT, wild type pre-S sequence. (B) Comparison of hepatitis B relapse free survival between patients with high (>10^6^ copies/mL; dashed line) and low (≤10^6^ copies/mL; solid line) viral loads. (C) Comparison of hepatitis B relapse free survival between patients with (dashed line) and without (solid line) LFpreSDel mutation. (D) Comparison of hepatitis B relapse free survival between patients with (dashed line) and without (solid line) basal core promoter A1762T/G1764A mutation. (E) Comparison of hepatitis B relapse free survival between patients with (dashed line) and without (solid line) precore G1896A mutation.

The genotypic features of HBV were assessed using preoperative serum samples. Because of low viral loads and undetectable HBV-DNA levels in some patients, the results of genotype, large fragment pre-S deletion (LFpreSDel), short fragment pre-S deletion, basal core promoter A1762T/G1764A mutations, and precore G1896A mutation were obtained in 140 (93.3%), 136 (90.0%), 135 (90.0%), 113 (75.3%), and 113 (75.3%) patients, respectively. Kaplan-Meier analysis was performed for these factors (Table S4). It was found that a preoperative viral load ≧10^6^ cps/ml (*P* = 0.004) and the presence of LFpreSDel (*P* = 0.003) correlated significantly with hepatitis B relapse (Figure 2A to C). Viral genotypes and basal core promoter A1762T/G1764A mutations were not found to be associated with hepatitis B relapse (Figure 2D). The presence of precore G1896A mutation was borderline associated with a shorter hepatitis B relapse-free survival (*P* = 0.064; Figure 2E).

**Figure 3:**
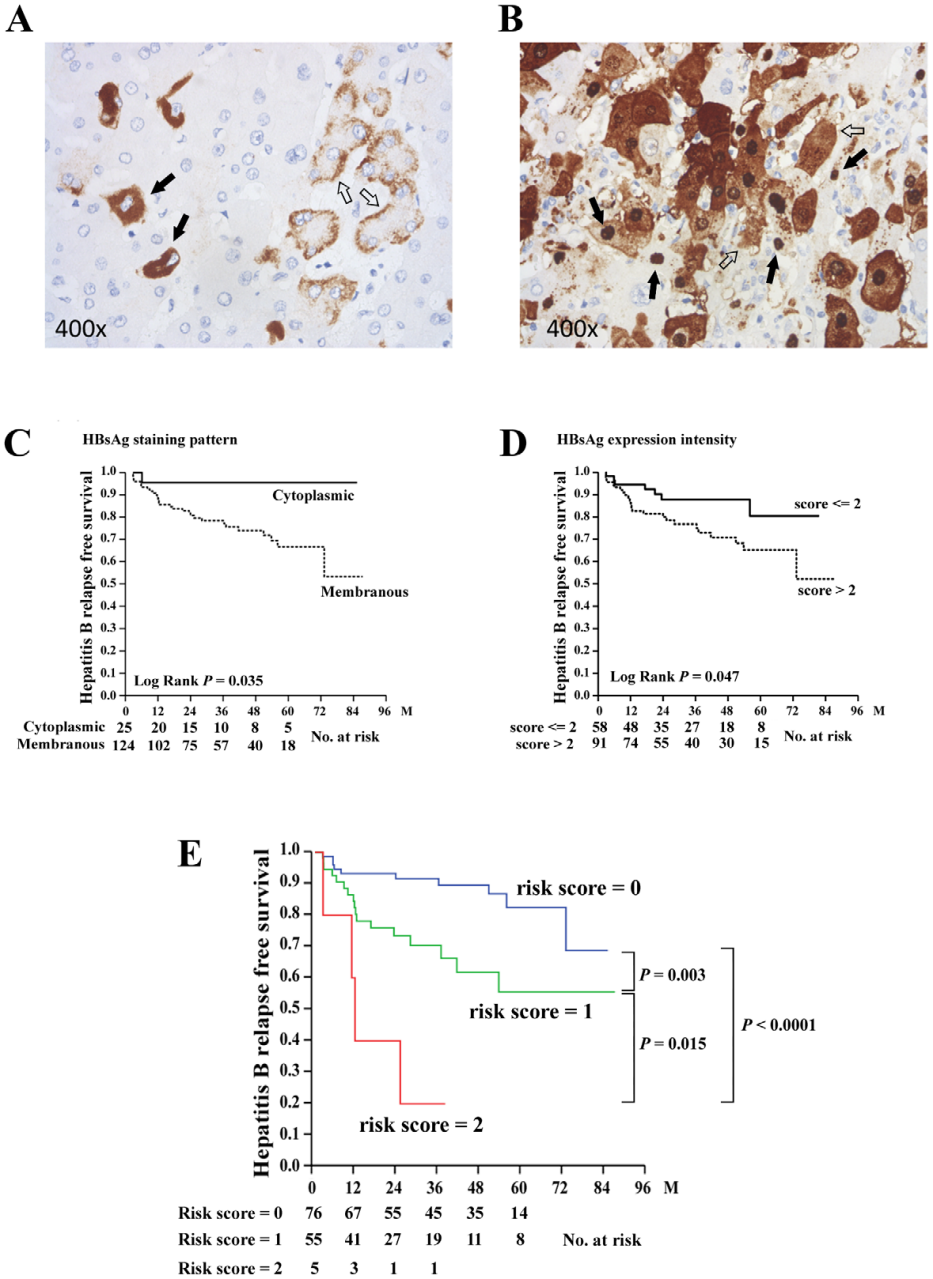
Immunohistochemical analysis of the explanted livers and risk score analysis. (A) Immunohistochemical analysis of HBsAg. Solid arrow, cytoplasmic staining pattern; Empty arrow, membranous staining pattern. (B) Immunohistochemical analysis of HBcAg. Solid arrow, nuclear HBcAg; Empty arrow, cytoplasm HBcAg. (C) Comparison of hepatitis B relapse free survival between patients with different staining patterns of HBsAg. Solid line, cytoplasmic pattern; Dashed line, membranous patter. (D) Comparison of hepatitis B relapse free survival between patients with different staining intensities of HBsAg. Solid line, intensity score ≤2; Dashed line, intensity score >2. (E) Combination of two independent risk factors (viral load and LFpreSDel) to predict hepatitis B relapse free survival after LT. Blue line, risk score = 0; Green line, risk score = 1; Red line, risk score = 2.

Immunohistochemistry analysis was performed to detect tissue expression of HBsAg (Figure 3A) and HBcAg (Figure 3B). All explanted livers were submitted for Ishak score assessment and immunohistochemistry analysis except for one sample, in which the explanted liver tissue was presented with massive tissue necrosis, preventing accurate evaluation. The staining patterns and intensities were categorized and the hepatitis B relapse free survivals were compared (Table S4). The results showed that the membranous staining pattern (*P* = 0.035) and high expression intensity (*P* = 0.047) of HBsAg in the explanted livers were associated with hepatitis B relapse (Figure 3C and D). However, no association could be found between HBcAg expression and hepatitis B relapse (Table S4).

To gain more insight into understanding the histopathological changes in the explanted livers from patients with high (≧10^6^ cps/ml) and low (<10^6^ cps/ml) serum levels, the histopathological characteristics were compared. Patients with high viral load had a higher Ishak score (*P* = 0.005; attributive to focal necrosis and portal inflammation) as well as a higher percentage of nuclear (*P* = 0.004) and cytoplasmic (*P*<0.0001) HBcAg expression. In contrast, when compared between patients with and without LFpreSDel, it was found that the presence of LFpreSDel mutation was associated with a lower Ishak score (P = 0.003; attributive to periportal inflammation and necrosis). Of note was that neither large nor short fragment pre-S deletion was associated with the staining patterns or expression intensities of tissue HBsAg in this study. The presence of precore G1896A mutation was associated with higher expression intensities of HBsAg (*P* = 0.035) and confluent necrosis (*P* = 0.030) in the explanted liver tissues. The presence of basal core promoter mutations was borderline associated with a higher percentage of cytoplasmic HBcAg expression (P = 0.048).

### Identification of independent predictors for hepatitis B relapse

Five factors significantly associated with hepatitis B relapse free survival (by Kaplan-Meier analysis) were included for multivariate analysis using Cox proportional hazard model. They were duration of preoperative LAM usage ≤3 M, preoperative viral load ≧10^6^ cps/ml, presence of LFpreSDel mutation, membranous staining pattern of HBsAg, and high expression intensity of HBsAg. After adjusting for other confounding factors, only the presence of LFpreSDel (HR, 3.766 [1.735–8.176], *P* = 0.001) and viral load ≧10^6^ copies/mL (HR, 2.368 [1.127–4.977], *P* = 0.023) were independent predictors for hepatitis B relapse (Table 1).

**Table 1:** Multivariate Cox proportional hazard regression analysis of the clinicopathologial and virological parameters for hepatitis B relapse free survival after LT.

Parameter	Comparison	Hazard ratio (95% CI)	*P* value
Duration of preoperative LAM treatment	>3 M	1	
	≤3 M	2.989 (0.702–12.723)	0.138
Predominant HBsAg staining pattern	Cytoplasmic	1	
	Membranous	3.249 (0.409–25.799)	0.265
HBsAg staining intensity (score 1 to 4)	0–2	1	
	3–4	2.070 (0.664–13.882)	0.152
Preoperative viral load	<10^6^ cps/mL	1	
	≧10^6^ cps/mL	2.368 (1.127–4.977)	0.023
LFpreSDel (>100 bp)	Absence	1	
	Presence	3.766 (1.735–8.176)	0.001

Of the 150 patients included, LFpreSDel mutation data was obtained in 136 patients. In these patients, hepatitis B relapse occurred in 4 of 5 (80.0%) patients carrying both high HBV-DNA levels (≧10^6^ copies/mL) and LFpreSDel mutations; in 17 of 55 (30.9%) patients carrying only one of the two conditions; and in 10 of 76 (13.2%) patients with neither condition. Kaplan-Meier analysis identified three subgroups with significantly different hepatitis B relapse free survival (Figure 3E). Four of 5 patients with two risk factors experienced hepatitis B relapse within two years after LT. In patients with only one risk, the cumulative relapse rates were 15.6% in the first year, 29.7% in the third year, and 44.4% in the fifth year, respectively. However, in patients without any risk, the cumulative relapse rates in the first, third, and fifth year were 6.7%, 8.4%, and 17.6%, respectively. Finally, of the 136 patients analyzed, 17 (12.5%) patients experienced reappearance of both HBsAg and HBV-DNA. Of these patients, 2 of 5 (40.0%), 10 of 55 (18.2%), and 5 of 76 (6.6%) patients, respectively, belonged to the three aforementioned subgroups.

## Discussion

Prophylactic administration of HBIG and preoperative usage of nucleos(t)ide analogs followed by life-long therapy are regarded as standard treatments for LT in HBV related end stage liver diseases. However, with an increasing number of approved oral antiviral agents, such as entecavir or tenofovir [26], it is now arguable whether LAM remains the first choice of virus-suppressing drugs for these patients. Furthermore, the dosage and duration for HBIG administration become disputable since potent life-long antiviral agents are now available. High dose or long-term HBIG costs expensively and a schedule of frequent injections is very inconvenient for patients. More and more recent investigation have reported the withdraw of long-term HBIG in patients receiving maintenance nucleos(t)ide analogue and outcome seems good especially in low risk patients [27], [28]. Due to financial consideration and limitation of health insurance policy, we adopted a short-term HBIG prophylaxis protocol in combination with life-long LAM therapy in our transplantation center. The prophylaxis protocol using short-term HBIG and life-long LAM therapy has also been reported by Nath et al [29]. In a mean follow-up period of 2 years, one of 14 patients remained HBsAg-positive but had normal liver function. In our cohort with more enrolled patients and longer follow-up period, however, the overall HBV relapse rate was quite high in comparison with other series applying high dose or long term HBIG. To address this issue, we performed this retrospective study to understand the limitation of adapting this preventive strategy.

As shown in the literature, positive HBV-DNA or high preoperative viral load before LT has always been identified as an important predictor for hepatitis B relapse after LT [18], [19]. In consistent with previous studies, the present data indicated that preoperative viral load greater than 10^6^ cps/mL was an independent risk factor for hepatitis B relapse after LT. In this study, however, an even stronger predictor was identified, which is the presence of LFpreSDel mutation. The pre-S region of HBV large surface protein mediates many essential functions in HBV life cycle, including virion morphogenesis and maturation, formation of secretory filamentous surface particles, and presentation of the putative receptor attachment site. Lines of evidence also indicated that this region contained important immunogenic epitopes, associated with effective host immune responses. Pre-S deletion was found in several HBV related chronic liver diseases and hepatocellular carcinoma [30], [31]. This kind of mutations has long been suspected to associate with hepatocarcinogenesis.

Our recent study showed that pre-S deletions could be further divided into two types, one with short fragment (<100 bp) deletion and the other with large fragment deletion (>100 bp) [24]. Sequencing data from previous studies showed that short fragment pre-S deletions were usually in-frame and located in a region surrounding the pre-S2 initiation codon, leaving the carboxyl 2/3 portion of the pre-S2 region largely intact. On the other hand, the positions of LFpreSDels were poorly defined and randomly distributed in the whole pre-S region. Conceivable, the immunogenic epitopes in the pre-S2 region had a better chance to be preserved in the short fragment pre-S deletion mutants compared with the LFpreSDel mutants. Previously, the short but not the large pre-S deletion was found to associate with postoperative recurrence of HCC. In the present study, we found that the large but not the short fragment pre-S deletion was associated with hepatitis B relapse post LT. Interestingly, a strong association was found between the present of LFpre-SDel and a lower Ishak histological score in the explanted livers, suggesting an immune escape like mechanism. In light of previous findings that important immunogenic epitopes were located in the pre-S2 region, which could be interrupted by LFpreSDel, we speculated that recipients infected with LFpreSDel mutants were less likely to be protected by HBIG prophylaxis. Therefore, a higher risk of hepatitis B relapse was found in such patients. Similarly findings have been reported by Grottola et al when evaluating the pre-S/S region of 14 HBV-positive candidates for LT, in whom hepatitis B relapse occurs after LT in 9 of them [32]. They found that the number of nucleotide mutations in the pre-S2 region was significantly higher in patients with hepatitis B relapse, compared with that in patients without HBV relapse. It is possible that extensive modification of pre-S2 protein (or develop of LFpreSDel in our cases) leads to conformational change, interfering with the viral envelopment and secretion processes. As a result, the mutant viruses re-infecting the donor liver tend to accumulate inside the hepatocytes, contributing to the failure of HBIG prophylaxis.

In the univariate analysis, there were two histopathologic factors (membranous HBsAg staining pattern and high HBsAg staining intensity) significantly associated with HBV relapse free survival. Unfortunately, neither of them constituted an independent predictor in multivariate analysis. Despite that, in medical facilities where analysis of HBV virological factors was not feasible, these histopathologic factors can be used as surrogate predictors. In fact, membranous HBsAg staining pattern and high HBsAg staining intensity could imply a high level of serum HBV-DNA.

Genotypic features of HBV, such as viral genotypes and mutations, were strongly associated with HBV pathogenesis as well as the pre- and post- LT clinical outcomes [33], [34], [35]. To our knowledge, the present study was the largest series extensively evaluated the impact of HBV virological characteristics on viral relapse after LT. In contrast to pre-S deletions, the other virological factor, such as precore/core variants, was not found to be associated with hepatitis B relapse in this study. Similar findings were reported by Lo et al and Gaglio et al, indicating that precore/core mutations did not influence hepatitis B relapse or outcome [35], [36]. On the other hand, the viral genotype has been reported to have impact on patient's outcome in LT [35]. In view of HBV relapse after LT, Lo et al reported that the cumulative rate of viral breakthrough due to LAM-resistance at 3 years was 4% for genotype B and 21% for genotype C (*P* = 0.017). However, Gaglio et al reported one of 8 (12.5%) patients with genotype B had HBV relapse compared to one of 18 (5.5%) patients with genotype C. Our data also supported that genotype did not influence the outcome of HBV relapse after LT.

The major concern in the presented study is the relatively high rate of HBV relapse after LT. In this study, hepatitis B relapse was defined as reappearance of HBsAg. As such, 33 over 150 patients (22%) met the criteria during a median follow-up period of more than 3 years. However, if a more stringent definition was adopted, such as reappearance of both HBsAg and HBV-DNA, only 12% of our patients (18 patients) were considered hepatitis B relapse. The high incidence of HBV relapse might be related to our anti-viral prophylaxis. In the present study, only short-term HBIG was used during the first week after LT. The viral DNA was not detected initially because viral replication was effectively inhibited by LAM. Fifteen of 33 patients with HBsAg relapse remained HBV-DNA negative at the end of follow-up with normal liver function. Unfortunately, prolonged usage of LAM resulted in drug resistance in 15 of 18 HBV-DNA relapse patients. According to the presented study, two independent risks were identified (high viral load and LFpreSDel mutation) for HBV relapse after LT. As such, only 6.6% of our patients in the low risk group experienced hepatitis B relapse, suggesting the current strategy might be adequate for the low risk patients. However, more aggressive prophylactic protocol should be applied for those patients with one or more risks, such as long-term HBIG [37] or replacement of LAM by Entecavir [26]. Additionally, with the availability of a more potent and state-of the-art antiviral regimen, such as tenofovir or entecavir, the need for continuous HBIG-prophylaxis is heavily challenged.

In conclusion, by analyzing preoperative clinical, virological and histopathological factors, we discovered that high viral load and LFpreSDel mutation were two independent predictors for hepatitis B relapse after LT. In patients presented with neither risk factor, it might be adequate to use short-term HBIG and life-long LAM prophylaxis, whereas more aggressive prophylactic strategy should be considered if patients were presented with one or both risk factors.

## Supporting Information

Tables S1 **Clinical characteristics and laboratory data for patients included.**
(DOC)

Table S2 Immunosuppression, rejection and co-infection* information.
(DOC)

Table S3 **Kaplan-Meier analysis of preoperative clinical parameters for hepatitis B relapse in patients receiving LT.**
(DOC)

Table S4 **Kaplan-Meier analysis of histopathological and virological factors for hepatitis B relapse in patients receiving LT.**
(DOC)
