# Supplementary material for: Large Fragment Pre-S Deletion and High Viral Load Independently Predict Hepatitis B Relapse after Liver Transplantation
Source: PLoS One. 2012 Feb 21;7(2):e32189. doi: 10.1371/journal.pone.0032189 (PMC3283733; doi:10.1371/journal.pone.0032189)
Supplement: Tables S1 — Clinical characteristics and laboratory data for patients included. (DOC) [file pone.0032189.s001.doc]

Table S1. Clinical characteristics and laboratory data for patients included.

| Clinical parameter |  | | Total | Male | Female | *P* |
| --- | --- | --- | --- | --- | --- | --- |
|  |  | | (N=150) | (N=127) | (N=23) |  |
| Recipient Age (years) |  | | 50.82±8.49 | 49.89±7.96 | 55.96±9.62 | **0.001** |
| Indication for transplantation | Advanced cirrhosis with or without HCC | | 123 (82.0%) | 107 | 16 | 0.235 |
|  | Acute on chronic liver failure | | 20 (13.3%) | 15 | 5 |  |
|  | Fulminant hepatitis | | 7 (4.7%) | 5 | 2 |  |
| HCC | Yes | | 72 (48.0%) | 60 | 12 | 0.821 |
|  | No | | 78 (52.0%) | 67 | 11 |  |
| Co-HCV infection | Yes | | 17 (11.3%) | 10 | 7 | **0.006** |
|  | No | | 133 (88.7%) | 117 | 16 |  |
| Alcohol | Yes | | 10 (6.7%) | 10 | 0 | 0.362 |
|  | No | | 140 (93.3%) | 117 | 23 |  |
| BUN (mg/dL) |  | | 21.77±21.19 | 20.34±18.69 | 29.43±30.81 | 0.183 |
| Creatinine (mg/dL) |  | | 1.26±1.20 | 1.25±1.12 | 1.33±1.58 | 0.756 |
| Total bilirubin (mg/dL) |  | | 7.79±10.51 | 7.58±10.62 | 8.93±10.07 | 0.575 |
| AST (U/L) |  | | 84.35±78.40 | 87.41±84.12 | 67.87±29.93 | **0.049** |
| ALT (U/L) |  | | 62.22±73.96 | 65.09±78.83 | 46.90±36.05 | 0.281 |
| ALK-P (U/L) |  | | 122.40±68.31 | 122.33±64.16 | 122.77±89.62 | 0.978 |
| Albumin (g/dL) |  | | 2.94±0.69 | 2.94±0.69 | 2.92±0.74 | 0.866 |
| Platelate (×1000/L) |  | | 69.13±43.06 | 66.19±41.31 | 84.74±49.48 | 0.058 |
| INR |  | | 1.82±0.64 | 1.82±0.67 | 1.84±0.47 | 0.871 |
| Child-Pugh score |  | | 9.33±2.35 | 9.35±2.41 | 9.26±2.09 | 0.873 |
| Child-Pugh class | A | | 24 (16.0%) | 21 | 3 | 0.830 |
|  | B | | 30 (20.0%) | 26 | 4 |  |
|  | C | | 96 (64.0%) | 80 | 16 |  |
| MELD score |  | | 19.39±7.85 | 19.21±7.64 | 20.39±9.04 | 0.510 |
| Donor age (years) |  | | 33.98±10.70 | 34.19±10.94 | 32.61±9.14 | 0.562 |
| Donor’ gender | Male | | 96 (64.0%) | 81 | 15 | 1.000 |
|  | Female | | 54 (36.0%) | 46 | 8 |  |
| Recipient HBeAg | Positive | | 17 (11.3%) | 16 | 1 | 0.473 |
|  | Negative | | 133 (88.7%) | 111 | 22 |  |
| Donor anti-HBc | Positive | | 52 (34.7%) | 43 | 9 | 0.640 |
|  | Negative | | 98 (65.3%) | 84 | 14 |  |
| Preoperative LAM treatment | Yes | | 79 (52.7%) | 68 | 11 | 0.655 |
|  | No | | 71 (47.3%) | 59 | 12 |  |
| Duration of preoperative LAM | ≧1 months | | 39 (26.0%) | 32 | 7 | 0.610 |
|  | ＜1 months | | 111 (74.0%) | 95 | 16 |  |
| Duration of Preoperative LAM | ≧3 month | | 29 (19.3%) | 25 | 4 | 1.000 |
|  | ＜3 month | | 121 (80.7%) | 102 | 19 |  |
| Operation related parameters | |  |  |  |  |  |
| Type of transplantation | | Deceased donor | 57 (38.0%) | 47 | 10 | 0.642 |
|  | | Living donor | 93 (62.0%) | 80 | 13 |  |
| GRWR | | >= 1 | 88 (58.7%) | 71 | 17 | 0.265 |
|  | | <1 and >= 0.8 | 49 (32.7%) | 44 | 5 |  |
|  | | < 0.8 | 13 (8.7%) | 12 | 1 |  |
| Operative time (min) | |  | 701.99±112.08 | 708.85±109.93 | 665.00±118.77 | 0.085 |
| Anhepatic time (min) | |  | 66.87±43.20 | 65.03±32.12 | 76.70±80.38 | 0.500 |
| Cold ischemic time (min) | |  | 259.08±276.38 | 264.26±275.26 | 225.53±289.81 | 0.593 |
| Warm ischemic time (min) | |  | 44.25±14.34 | 44.70±15.35 | 41.83±6.26 | 0.134 |
| Blood Loss (ml) | |  | 3638.21±455.40 | 3570.24±34364.05 | 4018.18±5582.55 | 0.672 |
| ICU admission day | |  | 17.42±14.58 | 16.70±13.89 | 21.39±17.78 | 0.156 |
| Hospitolization day | |  | 41.51±31.79 | 41.31±32.84 | 42.61±25.83 | 0.858 |
| Hospital Mortality | | Yes | 6 (4.0%) | 5 | 1 | 1.000 |
|  | | No | 144 (96.0%) | 122 | 22 |  |
| Acute rejection | | Yes | 10 (6.7%) | 9 | 1 | 1.000 |
|  | | No | 140 (93.3%) | 118 | 22 |  |
| Anti-HBs Antibody in 1M (IU/L) | |  | 223.64±217.70 | 224.65±216.49 | 218.66±229.93 | 0.916 |
| Anti-HBs Antibody in 3M (IU/L) | |  | 88.22±156.50 | 93.87±168.05 | 59.64±71.03 | 0.399 |
| Anti-HBs Antibody in 6M (IU/L) | |  | 14.33±39.50 | 14.09±40.95 | 15.67±31.00 | 0.860 |
| HBV relapse | | Yes | 33 (22.0%) | 31 | 2 | 0.108 |
|  | | No | 117 (78.0%) | 96 | 21 |  |

Parametric data were presented as mean ± standard deviation; Abbreviations: BUN, blood urine nitrogen; AST, aspartate aminotransferase; ALT, alanine aminotransferase; ALK-P, Alkaline phosphatase; LAM, Lamivudine; GRWR, graft to recipient body weight ratio.
