# Supplementary material for: Large Fragment Pre-S Deletion and High Viral Load Independently Predict Hepatitis B Relapse after Liver Transplantation
Source: PLoS One. 2012 Feb 21;7(2):e32189. doi: 10.1371/journal.pone.0032189 (PMC3283733; doi:10.1371/journal.pone.0032189)
Supplement: Table S2 — Immunosuppression, rejection and co-infection* information. (DOC) [file pone.0032189.s002.doc]

Table S2. Immunosuppression, rejection and co-infection* information

| Parameter | HBV relapse | Non-HBV relapse | *p* |
| --- | --- | --- | --- |
|  | (N=33) | (N=117) |  |
| Tarcolimus (FK506) through level† | 5.3±2.78 | 5.1±2.03 | 0.668 |
| Cellcept |  |  |  |
| Yes | 28 | 108 | 0.192‡ |
| No | 5 | 9 |  |
| Acute rejection |  |  | 0.692 ‡ |
| Yes | 1 | 9 |  |
| No | 32 | 108 |  |
| Treatment of rejection |  |  | 0.400 ‡ |
| Steroid bolus | 1 | 3 |  |
| Steroid recycles | 0 | 6 |  |
| Co-infection |  |  |  |
| Yes | 8 | 23 | 0.628‡ |
| No | 25 | 94 |  |
| Bacterial infection |  |  |  |
| Yes | 7 | 23 | 0.810‡ |
| No | 26 | 94 |  |
| Fungal infection |  |  |  |
| Yes | 3 | 9 | 0.727‡ |
| No | 30 | 108 |  |
| Cytomegalovirus infection |  |  |  |
| Yes | 5 | 8 | 0.161‡ |
| No | 28 | 109 |  |

* The data were collected within half year before the date of HBV relapse or last follow-up.

† Values are mean ± standard deviation and compared by t-test; ‡ Fisher’s exact test.
