# Supplementary material for: Large Fragment Pre-S Deletion and High Viral Load Independently Predict Hepatitis B Relapse after Liver Transplantation
Source: PLoS One. 2012 Feb 21;7(2):e32189. doi: 10.1371/journal.pone.0032189 (PMC3283733; doi:10.1371/journal.pone.0032189)
Supplement: Table S3 — Kaplan-Meier analysis of preoperative clinical parameters for hepatitis B relapse in patients receiving LT. (DOC) [file pone.0032189.s003.doc]

**Table S3.** Kaplan-Meier analysis of preoperative clinical parameters for hepatitis B relapse in patients receiving LT

| **Clinical parameter** | **No. of patients** | **Mean hepatitis B relapse-free survival (months) (95% CI)** | ***P**** |
| --- | --- | --- | --- |
| **A. Recipient’s factor:** |  |  |  |
| Recipient’s Age |  |  | 0.965 |
| ≦50 | 64 | 67.240(58.706-75.774) |  |
| ＞50 | 86 | 64.811(57.979-71.643) |  |
| Recipient’s gender |  |  | 0.256 |
| Female | 23 | 56.029(49.336-62.721) |  |
| Male | 127 | 66.181(59.848-72.515) |  |
| Indication for transplantation |  |  | 0.299 |
| Advanced liver cirrhosis with or without HCC | 123 | 65.748(59.182-72.134) |  |
| Acute on chronic liver disease | 20 | 69.300(58.681-79.920) |  |
| Fulminant hepatitis | 7 | 73.545(52.188-94.903) |  |
| HCC |  |  | 0.346 |
| No | 72 | 68.460(61.058-75.861) |  |
| Yes | 78 | 65.461(56.788-74.133) |  |
| Co-HCV infection |  |  | 0.239 |
| No | 133 | 68.627(62.518-74.736) |  |
| Yes | 17 | 46.262(35.508-57.015) |  |
| Alcohol |  |  | 0.998 |
| No | 10 | 67.512(61.364-73.659) |  |
| Yes | 140 | (63.111(40.581-85.641) |  |
| BUN (mg/dL) |  |  | 0.193 |
| ≦21 | 105 | 61.885(55.390-68.380) |  |
| ＞21 | 41 | 70.687(60.581-80.793) |  |
| Creatinine (mg/dL) |  |  | 0.248 |
| ≦1.2 | 107 | 61.887(55.403-68.371) |  |
| ＞1.2 | 39 | 72.279(62.837-81.721 |  |
| Total Bilirubin (mg/dL) |  |  | 0.795 |
| ≦1.3 | 29 | 69.304(56.828-81.780) |  |
| > 1.3 | 117 | 67.308(60.776-73.300) |  |
| AST (U/L) |  |  | 0.27 |
| ≦32 | 24 | 57.0.0(42.016-72.035) |  |
| ＞32 | 123 | 67.707(61.648-73-766) |  |
| ALT (U/L) |  |  | 0.175 |
| ≦36 | 63 | 58.821(48.980-68.663) |  |
| ＞36 | 83 | 67.311(60.815-73.806) |  |
| ALK-P (U/L) |  |  | 0.692 |
| ≦94 | 59 | 60.004(51.075-68.933) |  |
| ＞94 | 84 | 66.959(59.548-74.371) |  |
| Albumin (g/dL) |  |  | 0.869 |
| ≦3.5 | 121 | 68.021(61.564-74.477) |  |
| ＞3.5 | 27 | 54.478(44.696-64.260) |  |
| Platelate (×1000 /μL) |  |  | 0.136 |
| ≦150 | 137 | 66.098(59.409-72.786) |  |
| ＞150 | 8 | - |  |
| INR |  |  | 0.665 |
| ≦1.2 | 12 | 56.643(42.022-71.264) |  |
| ＞1.2 | 127 | 66.462(60.055-72.870) |  |
| Child-Pugh classification |  |  | 0.599 |
| A | 24 | 69.563(56.007-83.118) |  |
| B | 30 | 64.466(50.597-77.976) |  |
| C | 96 | 66.246(59.182-73.309) |  |
| MELD Score |  |  | 0.137 |
| ≦20 | 87 | 52.757(46.849-58.666) |  |
| ＞20 | 63 | 71.665(63.821-79.509) |  |
| Recipient HBeAg |  |  | 0.680 |
| Negative | 133 | 67.760(61.579-73.941) |  |
| Postive | 17 | 53.056(41.278-64.834) |  |
| **B. Donor’s factor:** |  |  |  |
| Donor’s Age (years) |  |  | 0.771 |
| ≦30 | 56 | 63.097(54.476-71.717) |  |
| ＞30 | 79 | 67.361(59.006-75.717) |  |
| Donor’s gender |  |  | 0.377 |
| Female | 51 | 69.675(60.095-79.255) |  |
| Male | 92 | 63.890(56.555-71.224) |  |
| Donor anti-HBc antibody |  |  | 0.088 |
| Positive | 52 | 63.523(56.023-71.023) |  |
| Negative | 98 | 67.023(60.372-73.675) |  |
| **C. Peri-operative factor:** |  |  |  |
| Preoperative LAM treatment |  |  | 0.307 |
| No | 71 | 63.496(55.281-71.711) |  |
| Yes | 79 | 66.329(56.539-76.118) |  |
| Duration of preoperative LAM |  |  | 0.058 |
| ≦1 month | 111 | 63.019(56.290-69.747) |  |
| ＞1 month | 39 | 78.834(70.957-86.710) |  |
| Duration of preoperative LAM |  |  | **0.040** |
| ≦3 months | 121 | 63.268(56.798-69.739) |  |
| ＞3 months | 29 | 81.692 (74.165-89.219) |  |
| Preoperative YMDD mutation |  |  | 0.161 |
| Yes | 8 | **-** |  |
| No | 142 | 66.484(60.368-72.599) |  |
| Type of Transplantation |  |  | 0.538 |
| Deceased donor | 57 | 64.116(55.243-72.990) |  |
| Living donor | 93 | 70.42(63.457-77.367) |  |
| GRWR |  |  | 0.339 |
| ≧1 | 88 | 67.856(60.542-75.169) |  |
| ＜1 and ≧0.8 | 49 | 64.458(54.318-74.597) |  |
| ＜0.8 | 13 | 73.249(73.249-73.249) |  |
| Blood loss (ml) |  |  | 0.538 |
| ＜1000 | 40 | 69.467(62.190-76.024) |  |
| ≧1000 | 110 | 59.673(49.201-70.144) |  |
| Biliary complication |  |  | 0.730 |
| no | 117 | 67.717(60.862-74.572) |  |
| yes | 33 | 62.989(51.090-74.888) |  |
| Acute rejection |  |  | 0.312 |
| no | 140 | 66.696(60.514-72.877) |  |
| yes | 10 | 67.167(57.595-76.375) |  |
| Anti-HBs antibody 1M (IU/L) |  |  | 0.813 |
| < 150 | 91 | 72.230(63.387-81.074) |  |
| ≧150 | 59 | 71.394(63.056-79.733) |  |
| Anti-HBs antibody 3M (IU/L) |  |  | 0.940 |
| < 50 | 100 | 72.584(64.257-80.893) |  |
| ≧50 | 50 | 70.854(62.404-79.304) |  |

* Kaplan-Meier survival analysis was performed using log rank test; BUN, blood urine nitrogen; AST, aspartate aminotransferase; ALT, alanine aminotransferase; ALK-P, Alkaline phosphatase; LAM, Lamivudine, GRWR, graft to recipient body weight ratio.
