# Supplementary material for: Large Fragment Pre-S Deletion and High Viral Load Independently Predict Hepatitis B Relapse after Liver Transplantation
Source: PLoS One. 2012 Feb 21;7(2):e32189. doi: 10.1371/journal.pone.0032189 (PMC3283733; doi:10.1371/journal.pone.0032189)
Supplement: Table S4 — Kaplan-Meier analysis of histopathological and virological factors for hepatitis B relapse in patients receiving LT. (DOC) [file pone.0032189.s004.doc]

**Table S4.** Kaplan-Meier analysis of histopathological and virological factors for hepatitis B relapse in patients receiving LT

| **Pathological and Virological Parameter** | **No. of patients** | **Mean HBV relapse-free survival (months) (95% CI)** | ***P*†** |
| --- | --- | --- | --- |
| **A. Pathological factor*** |  |  |  |
| Ishak Score | | |  |
| Total | | | 0.441 |
| ≦15 | 72 | 64.334(54.967-73.702) |  |
| ≧16 | 77 | 68.419(60.866-75.973) |  |
| Periportal or periseptal interface hepatitis | | | 0.731 |
| 0-2 | 92 | 67.371(59.987-74.754) |  |
| 3-4 | 57 | 66.878(57.505-76.250 |  |
| Confluent necrosis | | | 0.674 |
| 0-2 | 54 | 69.186(58.867-79.506) |  |
| 3-6 | 95 | 65.582(58.538-72.627) |  |
| Focal spotty necrosis, apoptosis, and focal inflammation | | | 0.957 |
| 0-2 | 131 | 67.863(61.518-74.208) |  |
| 3-4 | 18 | 58.959(46.920-70.997 |  |
| Portal inflammation | |  | 0.423 |
| 0-2 | 126 | 66.827(60.366-73.288) |  |
| 3-4 | 23 | 63.146(52.724-73.568) |  |
| Fibrosis and cirrhosis | | | 0.268 |
| 0-5 | 5 | - |  |
| 6 | 144 | 67.232(61.192-73.273) |  |
| HBsAg stain: Predominant expression pattern | | | **0.035** |
| Cytoplasmic | 25 | 81.715(74.821-88.609) |  |
| Membranous | 124 | 64.739(57.872-71.607) |  |
| HBsAg stain: Intensity of expression | | | **0.047** |
| 0 – 2 | 58 | 71.443(64.451-78.435) |  |
| 3 – 4 | 91 | 63.206(55.367-71.045) |  |
| HBcAg stain: Nuclear expression | | | 0.417 |
| 0 | 94 | 69.866(62.817-76.915) |  |
| 1 – 2 | 49 | 57.403(48.199-66.608) |  |
| 3 – 4 | 6 | 46.608(6.463-33.941) |  |
| HBcAg stain: Cytoplasmic expression | | | 0.254 |
| 0 | 89 | 70.762(63.624-77.899) |  |
| 1 – 2 | 44 | 55.819(46.035-65.603) |  |
| 3 – 4 | 16 | 55.288(40.707-69.869) |  |
|  |  |  |  |
| **B. Genotypic features of HBV** | | |  |
| Preoperative viral load | | | **0.018** |
| ＜10^6^ cps/ml | 111 | 70.049(63.907-76.191) |  |
| ≧10^6^ cps/ml | 39 | 57.308(43.994-70.622) |  |
| Genotype | | | 0.255 |
| B | 89 | 65.283(57.656-72.909) |  |
| C | 51 | 67.229(59.156-75.3020 |  |
| LFpreSDel (＞100bp) | | | **0.003** |
| Absence | 110 | 71.208(64.902-77.514) |  |
| Presence | 26 | 41.652(30.979-52.326) |  |
| Small fragment Pre-S deletion (＜100bp) | | | 0.832 |
| Absence | 117 | 67.291(60.780-73.802) |  |
| Presence | 18 | 49.946(40.007-59.884) |  |
| Basal core promoter A1762T/G1764A | | | 0.995 |
| Absence | 40 | 68.030(57.734-78.326) |  |
| Presence | 73 | 65.551(57.593-73.509) |  |
| Precore stop codon G1896A | | | 0.064 |
| Absence | 46 | 75.028(66.693-83.363) |  |
| Presence | 67 | 60.385(51.621-69.149) |  |

* One patient’s explanted liver presented with massive necrosis and was unable to provide accurate histological data; † Kaplan-Meier survival analysis was performed using log rank test;
